# Comparison of CTV_HR_ and organs at risk contours between TRUS and MR images in IB cervical cancers: a proof of concept study

**DOI:** 10.1186/s13014-020-01516-4

**Published:** 2020-04-06

**Authors:** Lucas C. Mendez, Ananth Ravi, Kevin Martell, Hamid Raziee, Yasir Alayed, Matt Wronski, Moti Paudel, Elizabeth Barnes, Amandeep Taggar, C. S. Wong, Eric Leung

**Affiliations:** 1 Department of Radiation Oncology, University of Toronto, Toronto, ON Canada; 2 Division of Radiation Oncology, London Health Sciences Centre, Western University, London, ON Canada; 3 Sunnybrook Health Sciences Centre, Toronto, ON Canada; 4 Division of Radiation Oncology, College of Medicine, King Saud University, Riyadh, Kingdom of Saudi Arabia

**Keywords:** Cervical cancer, Brachytherapy, Transrectal ultrasound

## Abstract

**Purpose:**

To compare CTV_HR_ and OAR dimensions and inter-rater agreement between magnetic resonance (MR) and trans-rectal ultrasound (TRUS) images in IB cervical cancer patients.

**Methods:**

IB cervical cancer patients treated with (chemo)radiotherapy plus MR-guided brachytherapy (BT) were prospectively enrolled in this study. Radiation oncologists contoured CTV_HR_ and OARs in pre-BT MR images (MRI) and intra-operative TRUS images. These contours were subsequently compared in regard to volume and dimension. Contour inter-rater agreement analysis was also investigated using kappa index (KI). Stata 15.0 was used for statistical analysis and a *p*-value < 0.05 was considered statistically significant.

**Results:**

TRUS CTV_HR_ volumes were statistically smaller than the respective MRI contoured volumes. TRUS CTV_HR_ thickness was found to be consistently smaller than MRI contours in all patients. No statistical difference was seen in width and height between the two different imaging modalities. MRI contours had a median KI of 0.66 (range: 0.56–0.77) while TRUS-based contours had a median KI of 0.64 (range: 0.47–0.77). Bladder and rectum had very satisfactory KI in both imaging modalities. Vaginal contours had moderate agreement in MR (0.52) and in TRUS images (0.58).

**Conclusion:**

TRUS images allow good visualization of CTV_HR_ and OARs in IB cervical cancer patients. Inter-rater contour variability was comparable between TRUS and MR images. TRUS is a promising modality on its own for image-guided BT.

## Introduction

Cervical cancer is endemic worldwide, affecting over half a million women every year according to the World Health Organization [1]. This is a particular major health problem in developing countries where 90% of cervical cancer deaths occur [1]. Multiple factors, such as ineffective screening programs and lack of broad community-based preventive initiatives are contributors to the poor outcomes seen in these countries. Likewise, treatment delays and limited access to modern technologies and even radiotherapy [2, 3] are essential elements for this poor prognosis.

Chemoradiation plus brachytherapy (BT) is the standard treatment modality for locally-advanced cervical cancer. Brachytherapy plays an important role in cervical cancer treatment, as survival and other oncological outcomes have been shown to be inferior whenever this is therapy omitted [4]. Over the two last decade, image-guided BT based on magnetic resonance (MR) images (MRI) has become the recommended standard technology for treatment of cervical cancer [5]. MRI has exceptional soft-tissue resolution that facilitates accurate delineation of tumour, and also allows satisfactory BT applicator reconstruction. However, MRI-based BT is resource and time-intensive and the workflow can result in up to 8 h of total procedure time per BT fraction [6]. Furthermore, the availability of MR scanners for BT planning are limited to a minority of centres worldwide.

Trans-rectal ultrasound (TRUS) may represent an alternative to MRI in image-guided BT for cervical cancer. TRUS has been used in prostate BT planning for decades and is known to have superior soft tissue contrast than computerized tomography [7]. Moreover, TRUS is less resource and time intensive than MRI. TRUS is also more convenient and may increase treatment precision, as imaging and treatment delivery could theoretically occur with patient under general anaesthesia and without any patient movement.

In this study, we investigated the role of TRUS-based BT planning. We compared TRUS-based contours in cervical cancer patients to the contours generated using standard MRI. Our hypothesis is that the CTV_HR_ and OARs can be delineated accurately on TRUS imaging.

## Materials & methods

Stage IB cervical cancer patients undergoing definitive (chemo)radiation and MRI based BT were enrolled in this prospective planning study. All IB patients that were seen by the main authors involved in this study were approached for study participation. Patients signed the informed consent approved by the institutional review ethics committee before participation in this study. All BT treatments were planned using MRI in accordance with GEC-ESTRO guidelines [8].

### Imaging acquisition and BT procedure

Patients received treatment between January 2017 and June 2018 at a single cancer centre. As per institutional protocol, all patients underwent a pre-BT MR scan of the pelvis prior to BT. In this current study, CTV_HR_ and OARs were contoured in these pre-BT MR images and compared with contours from the intra-procedural acquired TRUS images.

TRUS images were acquired in two consecutive BT fractions by one investigator only. At the time of BT, a detailed pelvic examination under anesthesia with the patient in low-dorsal lithotomy position was performed. During the BT procedure, the bladder was filled to a similar volume to the calculated bladder volume seen in the pre-BT MR scan. For that, an indwelling urinary catheter was inserted into the bladder followed by negative suction of the urine using a 60-mL syringe. Then, sterile saline was injected through the catheter to reproduce the pre-determined bladder volume. A rectal suction tube was used to clear rectal content followed by insertion of a 7 MHz transrectal 8848 BK ultrasound probe (12–4 MHz) (Analogic Corporation, Massachusetts, US). Para-axial images were captured every millimeter in the longitudinal axis using Oncentra Prostate (Elekta, Stocklholm, Sweden) and with the probe attached to a stepper unit. Images were acquired from the most cranial possible extent of the uterus to the level of the vaginal introitus. In three patients, the uterine fundus could not be captured due to anatomy limitation. There was no insurance that this image orientation was similar to MRI para-axial slices. MRI was performed on 1.5 Tesla (T) Ingenia MR-RT scanner (Philips, Amsterdam, The Netherlands) with a pelvic coil in the supine position. T2-weighted para-axial, para-sagittal and para-coronal images were obtained with 4 mm slice thickness, 0.4 mm spacing with an in-plan resolution of 0.43 mm × 0.43 mm.

### CTV_HR_ and OARs contouring

**Fig. 1:**
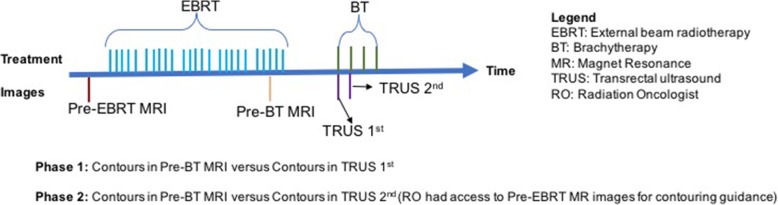
Chronologic diagram summarizing the relationship between MR scans and TRUS with EBRT and BT. EBRT: External beam radiotherapy; BT: Brachytherapy; MR: Magnet Resonance; TRUS: Transrectal ultrasound; RO: Radiation Oncologist

MRI and TRUS images were transferred to MIM® Software (Cleveland OH, US) after image acquisition. The contouring process was divided into two phases: In Phase 1, radiation oncologists (RO) contoured the CTV_HR_ and OARs, including bladder, rectum and vagina in both MR and TRUS image sets. TRUS images were typically acquired during the first fraction of BT were used in Phase 1. MR and TRUS images were contoured with a minimum gap of 7 days between them to minimize recall bias. In Phase 2, TRUS images typically acquired during the second BT insertion, were used. During this phase, ROs had direct access to the staging MR images (pre-EBRT) as a reference while contouring both MR and TRUS volumes (Fig. 1). All image set were acquired and contoured with no applicators in place.

CTV_HR_ was contoured as per GEC-ESTRO guidelines [8] and the cervix was called CTV-HR as only IB patients were included in this study. No specific instructions were given to RO regarding CTV-HR contouring. OARs were contoured as the whole organ including the outer wall and also following GEC-ESTRO guidelines. TRUS images had a limited field-of-view and did not capture the posterior rectal wall. Thus, only the anterior and lateral rectum wall were contoured in TRUS images. Gross tumour volume (GTV) was not evaluated in this study.

### Imaging evaluation

**Fig. 2:**
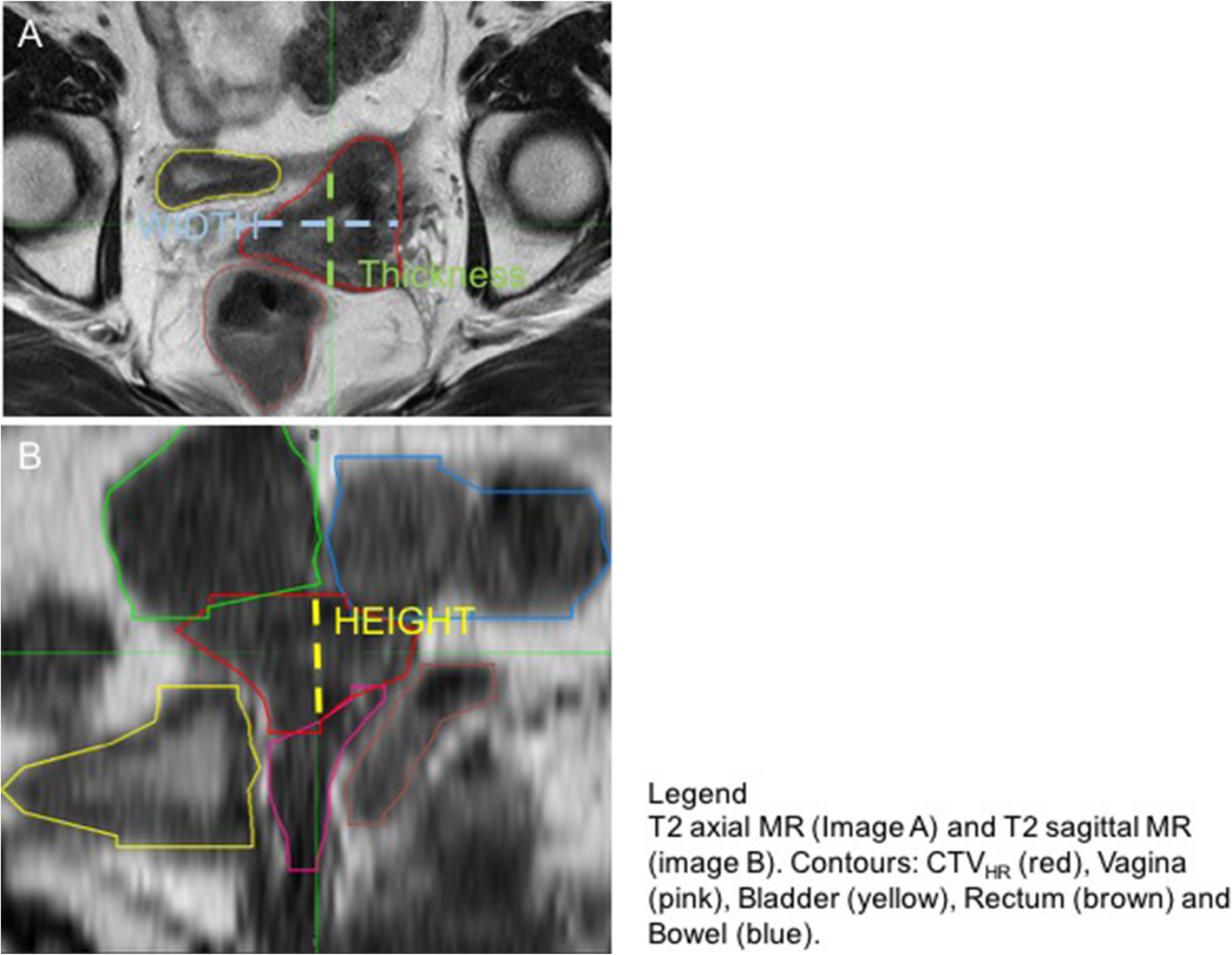
T2 axial MR (Image A) and T2 sagittal MR (image B). Contours: CTV_HR_ (red), Vagina (pink), Bladder (yellow), Rectum (brown) and Bowel (blue)

CTV_HR_ dimension and volume were directly compared between MRI and TRUS images. MIM® planning system automatically calculates a structure volume and this value was collected for both TRUS and MRI contours. Target dimensions were measured after localization of the mass centroid point, which is also automatically defined by MIM® software. Target thickness, width and height were measured based on the principal axes all contours, as exemplified in Fig. 2. These dimensions were measured in the axial, axial and sagittal plane, respectively.

**Fig. 3:**
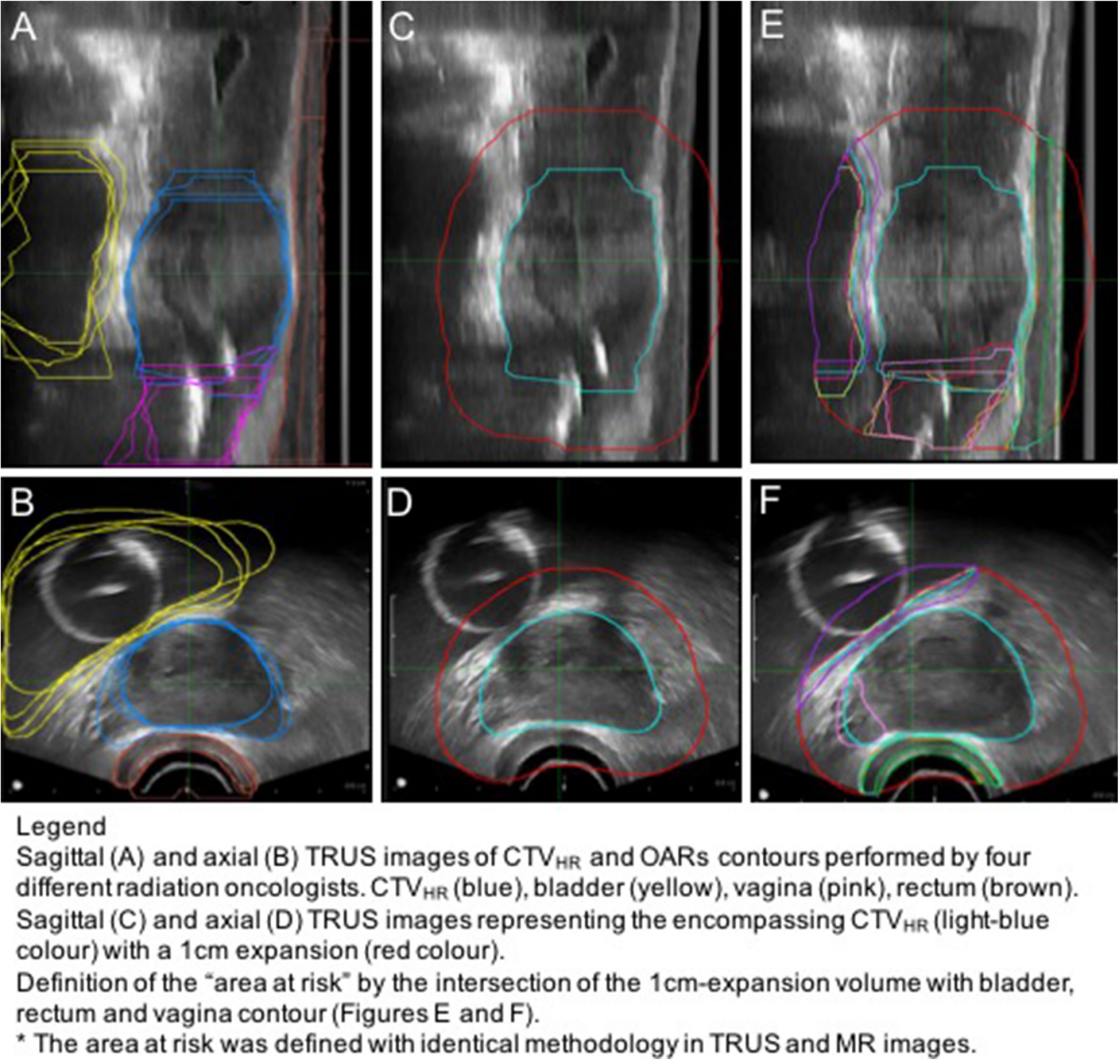
Diagram demonstrating the sequential steps involved in defining "areas at risk". Sagittal (**a**) and axial (**b**) TRUS images of CTV_HR_ and OARs contours performed by four different radiation oncologists. CTV_HR_ (blue), bladder (yellow), vagina (pink), rectum (brown). Sagittal (**c**) and axial (**d**) TRUS images representing the encompassing CTV_HR_ (light-blue colour) with a 1 cm expansion (red colour). Definition of the “area at risk” by the intersection of the 1 cm-expansion volume with bladder, rectum and vagina contour (Figures **e** and **f**). * The area at risk was defined with identical methodology in TRUS and MR images

Inter-rater contour variability was studied for both CTV_HR_ and OARs. Inter-rater agreement analysis for the target and OAR contours was conducted using the Computational Environment for Radiological Research (CERR) package in MATLAB (MathWorks, Massachusetts, US). Five ROs (one with 7 years, one with 4 years, two with with 3 years and one with 2 years of MRI-brachytherapy experience) and four ROs participated in contouring at Phase 1 and 2, respectively. The target contours, were consolidated into a single file and exported to CERR for analysis. Due to the steep dose gradients in BT, only the proximal OAR to the CTV_HR_ volume was evaluated. OAR contours were adjusted to only include regions that were inside the “area of risk”, defined by a 1 cm isometric margin expansion from the encompassing CTV_HR_ (union of all CTV_HR_ volumes) (Fig. 3). This strategy was also adopted as there are fundamental differences in field-of-views between MRI and TRUS images. Figure 3 shows in detail how the “area of risk” was defined.

### Statistical analysis

Descriptive analysis was used to summarize data on clinical characteristic, target volume and dimension. Wilcoxon signed-rank test was used to compare volumes and dimensions between MR and TRUS images. Target and OAR inter-rater contour variability was evaluated using kappa index (KI) and categorized in “almost perfect”, “substantial”, “moderate”, and “fair” agreement as described previously [9]. Stata 15.0 was used for statistical analysis and a *p*-value < 0.05 was considered statistically significant.

## Results

Five IB cervical cancer patients, out of eight approached patients, consented and were enrolled to participate in this study. Clinical characteristics are summarized in Table 1. All patients received EBRT with 45Gy in 25 fractions, followed by four fractions of MR-guided BT. One patient did not receive concomitant chemotherapy.

**Table 1:** Patients characteristics

					MRI Calculated Uterine Volume	Number of days post MR	
		Age	Stage	Uterus Position		First TRUS image	Second TRUS image
Patients	1	45	IB2	Anteroverted	71 mL	16	22
	2	92	IB1	Retroverted	39 mL	25	28
	3	35	IB2	Anteroverted	96 mL	4	7
	4	60	IB2	Anteroverted	85 mL	21	27
	5	41	IB2	Anteroverted	128 mL	21	25

**Fig. 4:**
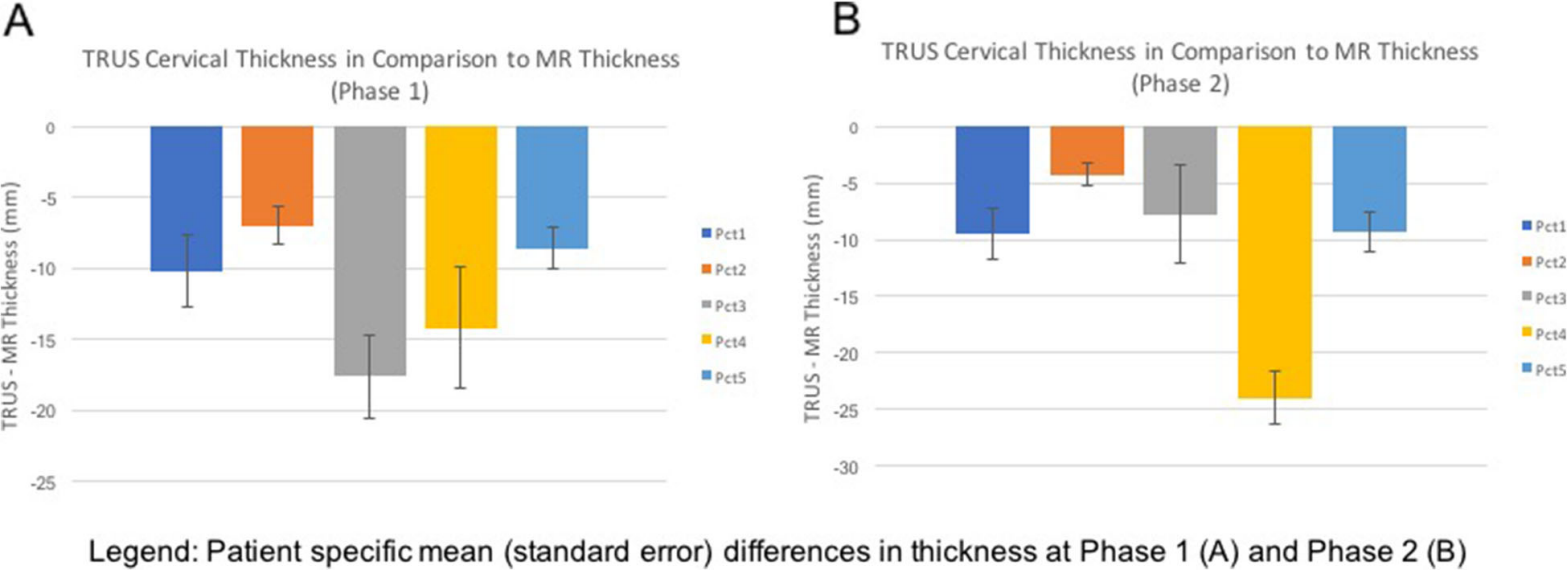
Patient specific diferences in target thickness on Phase 1 (**a**) and Phase 2 (**b**) (Mean and Standard Error)

Analysis of MRI and TRUS contours revealed that TRUS CTV_HR_ volumes were statistically smaller than the respective MRI contoured volumes (Table 2). The average difference between TRUS and MRI CTV_HR_ reduced from Phase 1 to Phase 2 (10 to 6 cc respectively) (Table 2) and the difference lost statistical significance in Phase 2. TRUS CTV_HR_ thickness was found to be consistently smaller than MRI contours in all patients (Fig. 4) and in both contouring phases. No statistical difference was seen in width and height between the two different imaging modalities.

**Table 2:** Average CTVHR dimension in MR and TRUS images in different contouring phases

	MRI 1st Phase	MRI 2nd Phase	TRUS 1st Phase	TRUS 2nd Phase
**HRCTV (SD)**	33 cc (5)*	31 cc (6)	23 cc (4)*	25 cc (4)
**Thickness (SD)**	37 mm (5)*	37 mm (6)#	26 mm (3)*	26 mm (3)#
**Height (SD)**	34 mm (8)	34 mm (6)	32 mm (5)	33 mm (6)
**Width (SD)**	44 mm (4)	41 mm (3)	40 mm (7)	41 mm (6)

* or #: *p* < 0.05 in Wilcoxon signed-rank test

Both TRUS and MRI CTV_HR_ overall inter-rater agreement was graded as “substantial”. A slight superiority was seen in MRI contours with a median kappa index of 0.66 (range: 0.56–0.77) while TRUS-based contours had a median index of 0.64 (range: 0.47–0.77). When the Kappa differences between MRI and TRUS were analyzed at separate time points, the mean difference between imaging modalities reduced from 0.06 at Time 1 to 0.01 at Time 2 (Table 3). Anecdotally, it was not possible to correlate KI with uterine volume, position or complete TRUS capture of the uterine fundus.

**Table 3:**
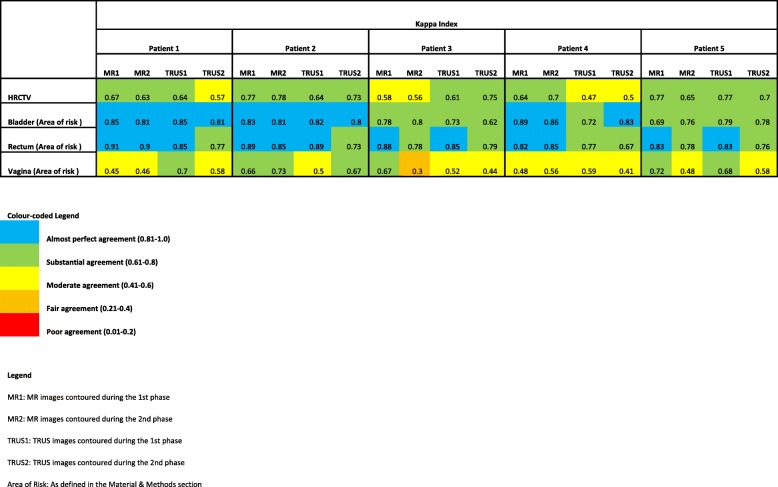
Calculated Kappa Index for CTVHR and OAR in MR and TRUS images at different phases

Organs at risk inter-rate volumetric agreement was also investigated (Table 3). For bladder, the Kappa index was 0.81 (range: 0.69–0.89) and 0.80 (range: 0.62–0.85) for MRI and TRUS contours, respectively. These Kappa indexes are categorized as “almost perfect” and “substantial” agreement for these imaging modalities. For the rectum contours, the Kappa index was 0.85 (range: 0.78–0.91), and 0.79 (range: 0.67–0.89) for MR and TRUS images, respectively. “Moderate” agreement between vaginal contours was seen for in both MR (0.52, range: 0.3–0.73) and TRUS images (0.58, range: 0.41–0.7).

## Discussion

Integration of volumetric imaging into gynecological BT has been transformational in the treatment of locally-advanced cervical cancers. With this technique, clinicians are capable of not only visualizing the pelvic anatomy but also appreciating the geometric relationship between target, OAR and the BT applicators. This technology has led to an improved understanding of the treatment volumes, dose-response characteristics of pelvic organs; ultimately, resulting in improvement of tumour control and toxicity [5, 10].

MRI scans have been used in the 3D-BT framework for more than two decades. MRI not only provides soft-tissue discrimination that is unparalleled by other imaging modalities, but it also allows for the reliable reconstruction of the implanted applicators. However, MR imaging also has some drawbacks: First, image acquisition can be lengthy as such internal organ motion can be problematic. Second, patients are required to be transferred from the operative bed to the MR scanner increasing treatment time and complexity. Last, the vast majority of cervical cancer cases occur in developing countries, where imaging cost and MR scanner scarce availability are significant limitations to adoption of 3D-BT [11].

TRUS also allows good soft-tissue definition and, in contrast to MRI, can be performed intra-operatively with the patient under anesthesia. Ultrasound machines are also more readily available when compared to MR scanners. Moreover, TRUS has been successfully adopted for image-guidance and treatment planning in prostate BT. In this context, this study investigates TRUS accuracy in defining pelvic volumes (CTV_HR_ and OARs) in patients with IB cervical tumours by comparing contours on TRUS imaging with contours on MR imaging.

Our results suggest that TRUS-defined CTV_HR_ are statistically smaller than MR CTV_HR_. This finding can be in part explained by the nature of TRUS acquisitions with the ultrasound probe compressing anteriorly the cervix. In agreement with this explanation, CTV_HR_ thickness was found to be consistently smaller on TRUS than on MR images. In a previous report, Schmid et al. also noted a smaller thickness in TRUS CTV_HR_ with a difference of approximately 3.5 mm between TRUS and MR contour in this dimension [12]. Difference in other CTV_HR_ dimensions (height and width) between MR and TRUS was not detected, and altogether, this is suggestive that differences in CTV_HR_ thickness is the primary driver for a systematic difference in target volume between imaging modalities.

TRUS CTV_HR_ in Phase 2 had on average less volumetric difference to MR than contours in Phase 1. This could possibly indicate a learning curve phase, since no specialized TRUS contouring training by the ROs was performed prior to this study. Access to pre-EBRT MR images by the ROs during Phase 2 is another possible explanation for less volumetric difference between TRUS and MRI CTV_HR_ contours.

To our knowledge, this is the first study to investigate inter-rater agreement between TRUS and MR images for both CTV_HR_ and OARs in cervical cancer patients. Surprisingly, CTV_HR_ contour agreement were rated as “substantial” with both imaging modalities (median Kappa index of 0.66 and 0.64 for MR and TRUS contours, respectively). Bladder and rectum contours in both MR and TRUS are very satisfactory, with “substantial” to “almost perfect” inter-rater agreement seen in this study. The methodology used for OARs comparison was based in the existent differences in nature between MR and TRUS images, as the posterior aspect of the rectum and the anterior part of the bladder are not or poorly visualized in TRUS images. Of note, dosimetric parameters associated with toxicity (like D0.1cc and D2cc) are found close to the CTV_HR_, and in our view, a 1 cm isometric expansion from the encompassed CTV_HR_ is appropriate for this analysis.

Together with prior publications [12, 13], the results here presented contribute to the effort in developing TRUS based BT planning in cervical cancers. In our view, the volumetric difference seen in CTV_HR_ for IB cervical cancers between TRUS and MR contours seem to more strongly associate with the fundamental difference in image acquisition between these two imaging modalities than by any inaccuracy of TRUS to visualize the target. The systematic difference in CTV_HR_ thickness and similar inter-rater agreement between imaging modalities suggest similar conclusion.

This study has limitations. First, has limited to compare TRUS and MR contours in patients with FIGO IB cervical cancers only. Prior publications have suggested that IB patients are less benefitted by image-guided brachytherapy than more advanced cancers in terms of local, once a great extent or all cancerous disease is already encompassed by 2D-based dose prescription. Nevertheless, TRUS-guided brachytherapy allows for good organ at risk visualization and may reduce toxicity in this population, as previously seen with CT or MRI-based brachytherapy [10]. Second, GTV visualization in TRUS images is challenging. Third, TRUS and MRI volume comparison was performed in image sets without applicator in situ. Last, the OARs contouring analysis used in this study has not yet been validated, although seems intuitive and logical due to the proximity of the hot spots to the CTV_HR_.

## Conclusion

TRUS images allow good visualization of CTV_HR_ and OARs in IB cervical cancer patients. Inter-rater contour variability was comparable between TRUS and MR images. TRUS is a promising modality on its own for image-guided BT.

## Data Availability

The datasets during and/or analyzed during the current study available from the corresponding author on reasonable request.

## Abbreviations

CTV_HR_: High-risk clinical target volume
OAR: Organs at risk
TRUS: Transrectal ultrasound
MR: Magnetic Resonance
MRI: Magnetic Resonance Images
KI: Kappa Index
BT: Brachytherapy
EBRT: External beam radiotherapy
RO: Radiation Oncologist
